# Quantitative analysis of studies that use artificial intelligence on thyroid cancer: a 20-year bibliometric analysis

**DOI:** 10.3389/fonc.2025.1525650

**Published:** 2025-03-18

**Authors:** YingZheng Gao, JiaHao Chen, Tao Fu, Yi Gu, WeiDong Du

**Affiliations:** The First Affiliated Hospital of Zhejiang Chinese Medical University (Zhejiang Provincial Hospital of Traditional Chinese Medicine), Hangzhou, China

**Keywords:** thyroid cancer, neoplasm, artificial intelligence, bibliometric analysis, cancer

## Abstract

In recent years, with the rapid advancement of computer science, artificial intelligence has found extensive applications and has been the subject of significant research within the healthcare industry, particularly in areas such as medical imaging, diagnostics, biomedical engineering, and health data analytics. Artificial intelligence has also made considerable inroads in the diagnosis and treatment of thyroid cancer. This study aims to evaluate the progress, current hotspots, and potential future directions of research on artificial intelligence in the field of thyroid cancer through a bibliometric analysis. This study retrieved literature on the application of artificial intelligence in thyroid cancer from 2004 to 2024 from the Web of Science Core Collection (WoSCC) database. A retrospective bibliometric analysis and visualization study of the filtered data were conducted using VOSviewer, CiteSpace, and the Bibliometrix package in R software. A total of 956 articles from 70 countries/regions were included. China had the highest number of publications, with Shanghai Jiao Tong University (China) being the most prolific research institution. The most prolific author was Wei, X. (n=14), while Haugen, B. R. was the most co-cited author (n=297). The Frontiers in Oncology (35 articles, IF=3.5, Q1) was the most frequently publishing journal, and Thyroid (cited 1,705 times) was the most co-cited journal. Keywords such as ‘ultrasound,’ ‘deep learning,’ and ‘diagnosis’ indicate research hotspots in this field. This study provides a comprehensive exposition of the current advancements, emerging trends, and future directions of artificial intelligence in thyroid cancer research. It serves as a valuable resource for clinicians and researchers, offering a systematic understanding of key focal areas in the field, thereby assisting in the identification and determination of future research trajectories.

## Introduction

Thyroid cancer ranks among the most prevalent endocrine malignancies worldwide (1). Although the prognosis for thyroid cancer is favorable, the incidence of this disease has been rising rapidly in recent years (2), and thyroid cancer ranks ninth in incidence among cancers worldwide (3, 4). The treatment and diagnosis of thyroid cancer have drawn increasing attention from scholars both domestically and internationally.

In recent years, with the rapid advancements in the field of computing, artificial intelligence has found numerous applications and been extensively studied within the healthcare industry, particularly in areas such as medical image processing, diagnostics, biomedical engineering, and health data analytics (5, 6). With the growing interest in the application of artificial intelligence (AI) in thyroid cancer research, a significant body of literature has emerged on the subject. By training models, AI has demonstrated the capability to identify and differentiate between thyroid cancer and benign nodules, thereby enhancing diagnostic accuracy and efficiency (7, 8). Through the use of training datasets, AI can automate the analysis of pathological slides, improving the precision of pathological diagnoses (9). Moreover, based on clinical data from thyroid cancer patients, AI can predict disease progression and the risk of recurrence (10, 11).

However, no bibliometric analysis has been conducted to systematically summarize the application of artificial intelligence in thyroid cancer research. Bibliometric analysis, as a method for characterizing research activities within a given field, provides a comprehensive and objective evaluation of literature data through mathematical and statistical approaches. This method reveals the current progress and emerging trends in the field, offering valuable insights into its ongoing development.

This paper presents the first bibliometric analysis in the field of artificial intelligence applications in thyroid cancer. It aims to comprehensively review the relevant literature from 2004 to 2024, offering insights into research progress, existing challenges, current focal points, and projections for future directions in this domain. The findings are intended to assist clinicians and researchers in systematically identifying key research priorities and guiding future investigations.

## Methods

### Database and search strategy

**Figure 1:**
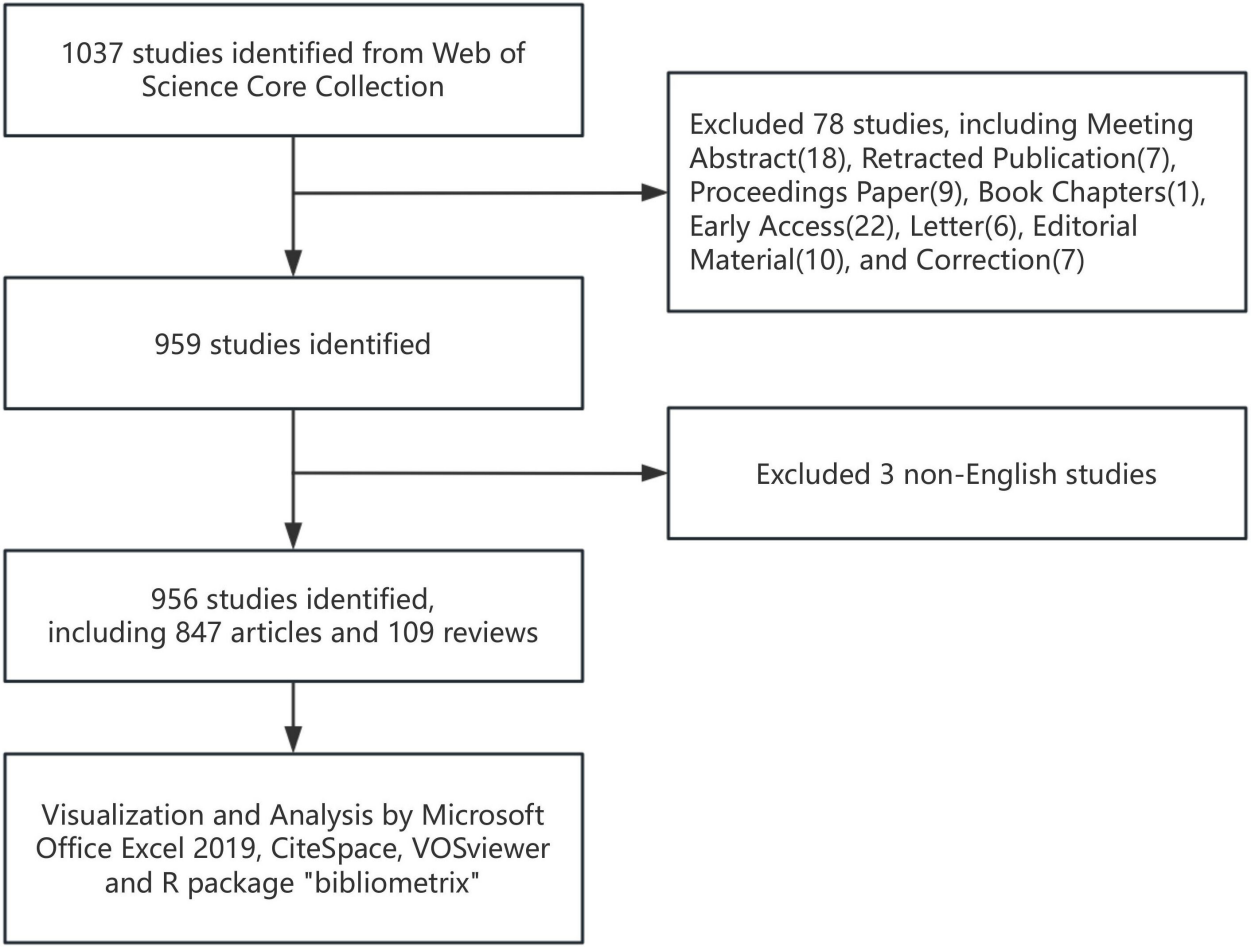
Flowchart of data collection.

Two independent authors conducted a literature search within the Web of Science Core Collection (WoSCC) database. The choice of this database was motivated by its recognition as the most authoritative and widely utilized resource for bibliometric analysis (12). The search was restricted to publications indexed in the Science Citation Index Expanded (SCI-EXPANDED) and the Social Sciences Citation Index (SSCI). The timeframe extended from August 1, 2004, to August 1, 2024. The search strategy was as follows: TS= (‘artificial intelligence’ OR ‘computational intelligence’ OR ‘machine learning’ OR ‘deep learning’ OR ‘feature learning’ OR ‘hierarchical learning’ OR ‘neural network’ OR ‘bayesian network’ OR ‘Supervised Learning’ OR ‘Unsupervised Learning’ OR ‘semantic segmentation’ OR ‘big data’ OR ‘Data mining’ OR ‘computer vision’) AND TS= (thyroid) AND TS= (‘cancer’ OR ‘neoplasm’ OR ‘tumor’ OR ‘malignancy’ OR ‘neoplasia’ OR ‘carcinoma’ OR ‘oncology’). The document types were limited to articles and reviews, and the publication language was restricted to English. The detailed retrieval process is shown in **Figure 1**.

### Data analysis

In this investigation, bibliometric analysis was performed utilizing VOSviewer (version 1.6.20), CiteSpace (version 6.2.R7), and the R package ‘bibliometrix’ (version 4.0.0), ensuring a comprehensive exploration of the relevant literature.

VOSviewer is a widely utilized software program in bibliometric analysis, facilitating data processing, visualization, and analysis. The visual representation is composed of nodes and links, where variations in color, size, and proximity of the nodes serve to convey significant information. In this study, VOSviewer was employed to visualize and analyze the relationships among authors, countries, institutions, journals, references, and keywords.

We employed CiteSpace to generate dual-map overlays of journals for comprehensive analysis, alongside conducting Citation Burst analyses. The dual-map overlays allowed us to distinguish the evolution of knowledge structures and identify emerging research frontiers. The Citation Burst analysis involved calculating the frequency and growth rate of cited references across various time periods to detect references that have rapidly gained prominence, thereby indicating a heightened focus on specific topics of interest.

Bibliometrix, an R-based open-source software tool, was utilized in this study to illustrate global trending topics.

Additionally, all quantitative analyses for the publications were conducted using Microsoft Office Excel 2019.

## Results

### Global trend of publications and citations

**Figure 2:**
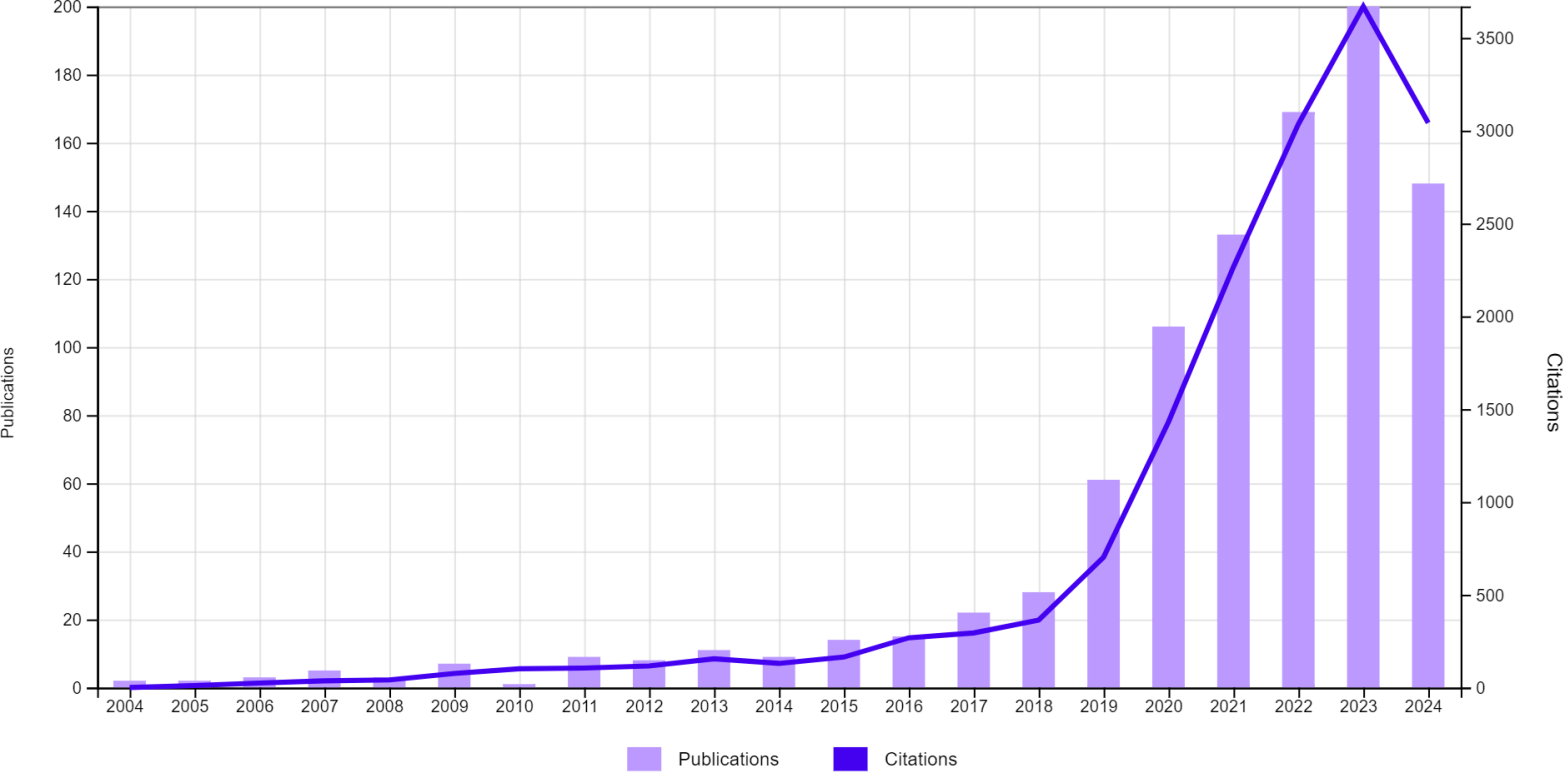
Global trends in the publication volume and citation frequency of articles related to artificial intelligence and thyroid cancer over the past two decades.

A total of 1,037 articles were retrieved from the Web of Science Core Collection (WoSCC). Following further screening (**Figure 1**), 956 publications on the topic of artificial intelligence in thyroid cancer were ultimately selected, including 847 original research articles and 109 reviews. From 2004 to 2024, the number of publications per year has shown a continuous upward trend (**Figure 2**). This rapid increase may be attributed to the swift advancements in artificial intelligence algorithms within the field of computer science, which have led to a significant rise in thyroid cancer-related research. It is anticipated that the number of newly published articles will continue to grow in the coming years.

### Country and institutional analysis

**Figure 3:**
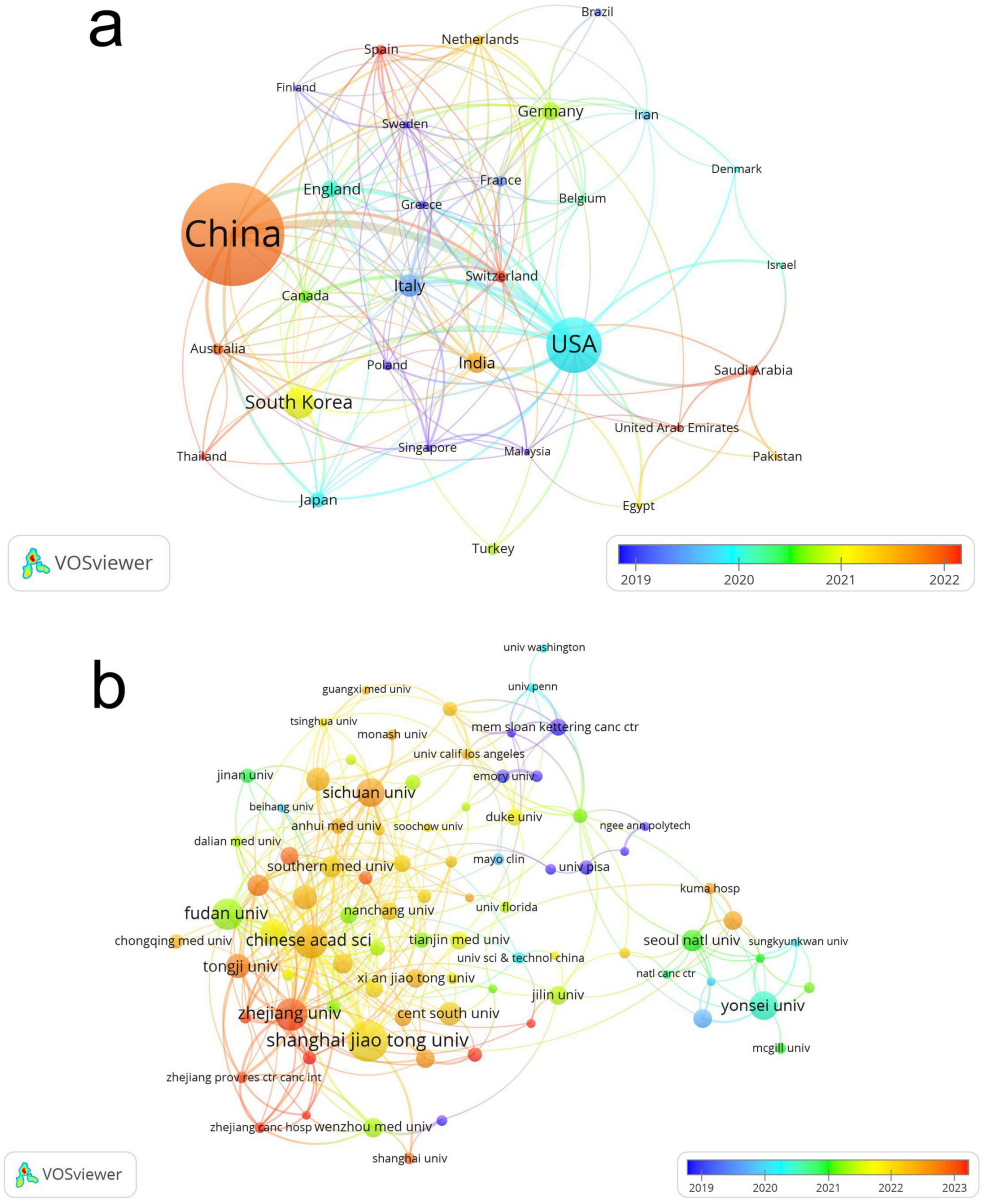
Visualization of research contributions by countries **(a)** and institutions **(b)** in the field of artificial intelligence and thyroid cancer.

A total of 70 countries, encompassing 1,617 institutions, have contributed to research on the application of artificial intelligence in thyroid cancer, with Asia emerging as the predominant contributor. Notably, two of the top three countries in terms of publication volume are from Asia, including China (n=486, 50.84%), which ranks first, and South Korea (n=83, 8.68%), which ranks third (**Table 1**). The United States, representing North America, ranks second (n=193, 20.19%). **Figure 3a** illustrates the degree of collaboration between countries with five or more publications. The size of the nodes reflects the volume of publications from each country, while the color of the nodes represents the years of higher publication activity. The thickness of the edges indicates the strength of collaboration between countries. China, despite its relatively late start in research on AI applications in thyroid cancer, has now surpassed other nations in publication output, with significant collaborations observed between China and the United States. Notably, the average number of citations per article in China is 13.7, whereas in the United States, this figure reaches 26.13. This disparity suggests that although China produces a high volume of publications, the research quality of these articles lags behind that of the United States.

**Table 1:** Top 10 countries and institutions on research of application of artificial intelligence in thyroid cancer.

Rank	Country	Counts	Citations	Institution	Counts
1	China (Asia)	486 (50.84%)	6,659	Shanghai Jiao Tong University (China)	38 (3.97%)
2	USA (North America)	193 (20.19%)	5,045	Chinese Academy of Sciences (China)	29 (3.03%)
3	South Korea (Asia)	83 (8.68%)	1,358	Zhejiang University (China)	27 (2.82%)
4	Italy (Europe)	50 (5.23%)	1,378	Fudan University (China)	26 (2.72%)
5	India (Asia)	43 (4.50%)	902	Sichuan University (China)	23 (2.41%)
6	Germany (Europe)	36 (3.77%)	900	Yonsei University (South Korea)	23 (2.41%)
7	England (Europe)	34 (3.56%)	1,018	Zhengzhou University (China)	20 (2.09%)
8	Japan (Asia)	29 (3.03%)	528	Huazhong University of Science and Technology (China)	18 (1.88%)
9	Canada (North America)	20 (2.09%)	465	Tongji University (China)	18 (1.88%)
10	Turkey (Europe/Asia)	19 (1.99%) ​	107	Central South University (China)	17 (1.78%)

**Table 1** summarizes the contributions of the ten institutions that have made the most significant research advancements in the application of artificial intelligence in thyroid cancer. The top three institutions are Shanghai Jiao Tong University (n=38, 3.97%), Chinese Academy of Sciences (n=29, 3.03%), and Zhejiang University (n=27, 2.82%). Notably, among the top ten institutions, nine are based in China, **Figure 3b** illustrates the collaborative relationships among the 87 institutions that have published more than five articles, revealing a robust intra-national collaboration within Chinese and South Korean institutions, while international cooperation appears to be relatively limited.

### Analysis of journals and co-cited journals

**Figure 4:**
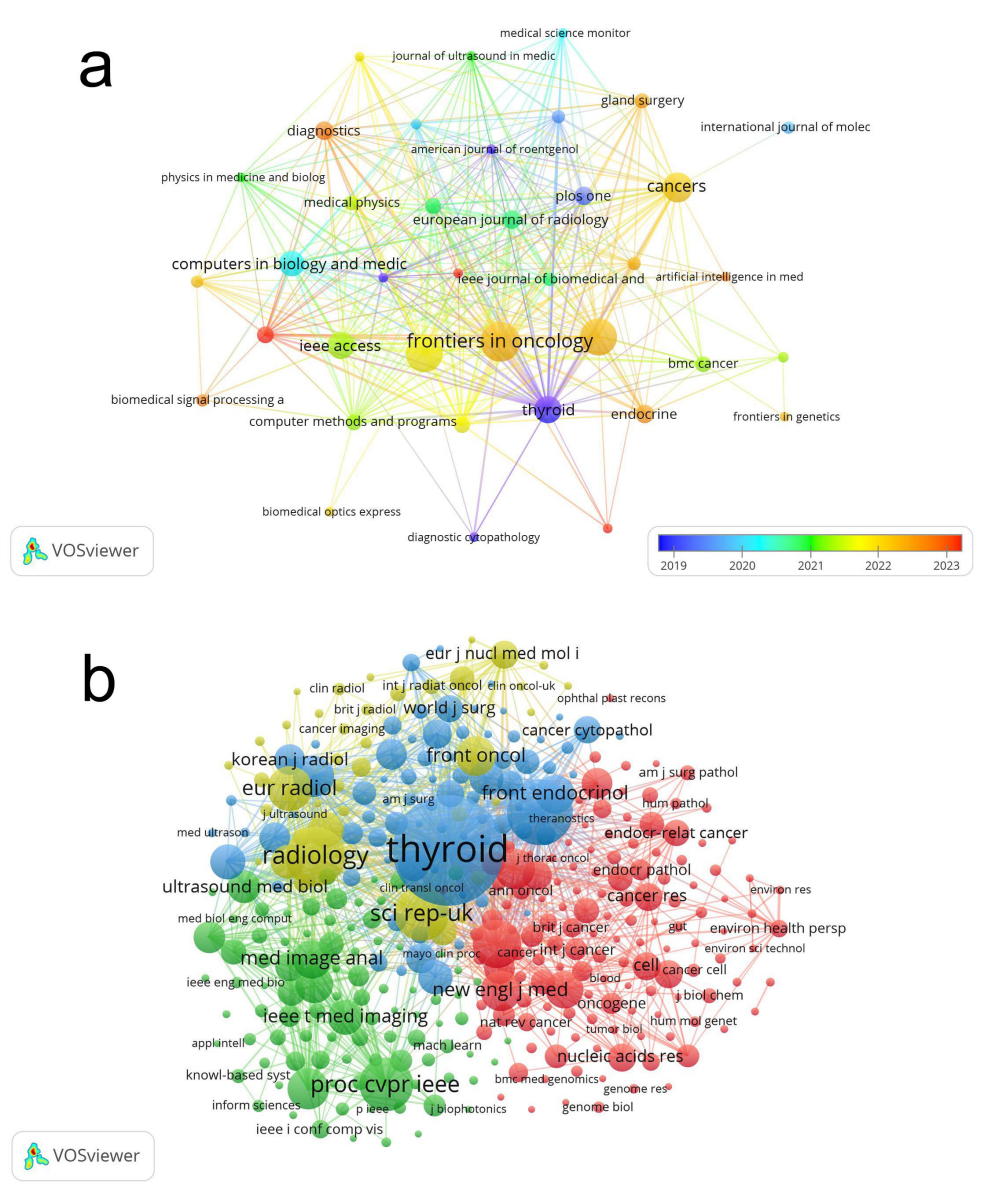
The visualization of journals **(a)** and co-cited journals **(b)** on research of application of artificial intelligence in thyroid cancer.

Over the past two decades (2004–2024), our search identified a total of 385 journals that have published studies on the application of artificial intelligence in thyroid cancer research. **Table 2** presents the top 15 journals, which account for more than 25% of the published articles. All of these journals are high-quality publications ranked in Q2 or above in the Journal Citation Reports (JCR). Among these, the *Frontiers in Oncology* (IF = 3.3, Q2) is the most prolific, having published 35 papers. This is followed by *Frontiers in Endocrinology* (IF = 3.9, Q2) and *Scientific Reports* (IF = 3.8, Q1). We utilized VOSviewer to visualize the journal citations analysis (**Figure 4a**). *Frontiers in Oncology* and *Frontiers in Endocrinology* have been cited most frequently from 2022 to 2023, with a large number of citations from journals such as *Thyroid*, *Scientific Reports*, and *Cancers*, etc.

**Table 2:** Top 15 journals and co-cited journals for research of application of artificial intelligence in thyroid cancer.

Rank	Journal	Count	IF	Q	Co-cited Journal	Co-citation
1	Frontiers in Oncology	35 (3.66%)	3.3	Q2	Thyroid	1,705
2	Frontiers in Endocrinology	32 (3.35%)	3.9	Q2	Journal of Clinical Endocrinology & Metabolism	733
3	Scientific Reports	32 (3.35%)	3.8	Q1	Radiology	637
4	Cancers	24 (2.51%)	4.5	Q1	Scientific Reports - UK	544
5	IEEE Access	21 (2.20%)	3.4	Q2	Proceedings of CVPR IEEE	536
6	Thyroid	21 (2.20%)	5.8	Q1	PLOS ONE	447
7	Computers in Biology and Medicine	19 (1.99%)	7	Q1	European Radiology	404
8	Diagnostics	12 (1.26%)	3	Q1	American Journal of Roentgenology	353
9	Endocrine	12 (1.26%)	3	Q2	Lecture Notes in Computer Science	346
10	European Radiology	12 (1.26%)	4.7	Q1	The New England Journal of Medicine	342
11	PLOS ONE	12 (1.26%)	2.9	Q1	Cancers	337
12	Quantitative Imaging in Medicine and Surgery	11 (1.15%)	2.9	Q2	Frontiers in Oncology	324
13	BMC Cancer	10 (1.05%)	3.4	Q2	Medical Image Analysis	315
14	Computer Methods and Programs in Biomedicine	10 (1.05%)	4.9	Q1	Frontiers in Endocrinology	310
15	European Radiology	10 (1.05%) ​	4.7	Q1	arXiv	302

By setting a minimum citation threshold of 20 for cited journals, we identified 356 journals to map the co-citation network (**Figure 4b**), illustrating the co-citation relationships between different journals. *Thyroid* has tight co-citation relationships with *Frontiers in Endocrinology*, and *Radiology*, etc. According to the data in **Table 2**, *Thyroid* ranked first with 1,705 co-cited references, followed by *Journal of Clinical Endocrinology & Metabolism* (co-citations = 733) and *Radiology* (co-citations = 637).

**Figure 5:**
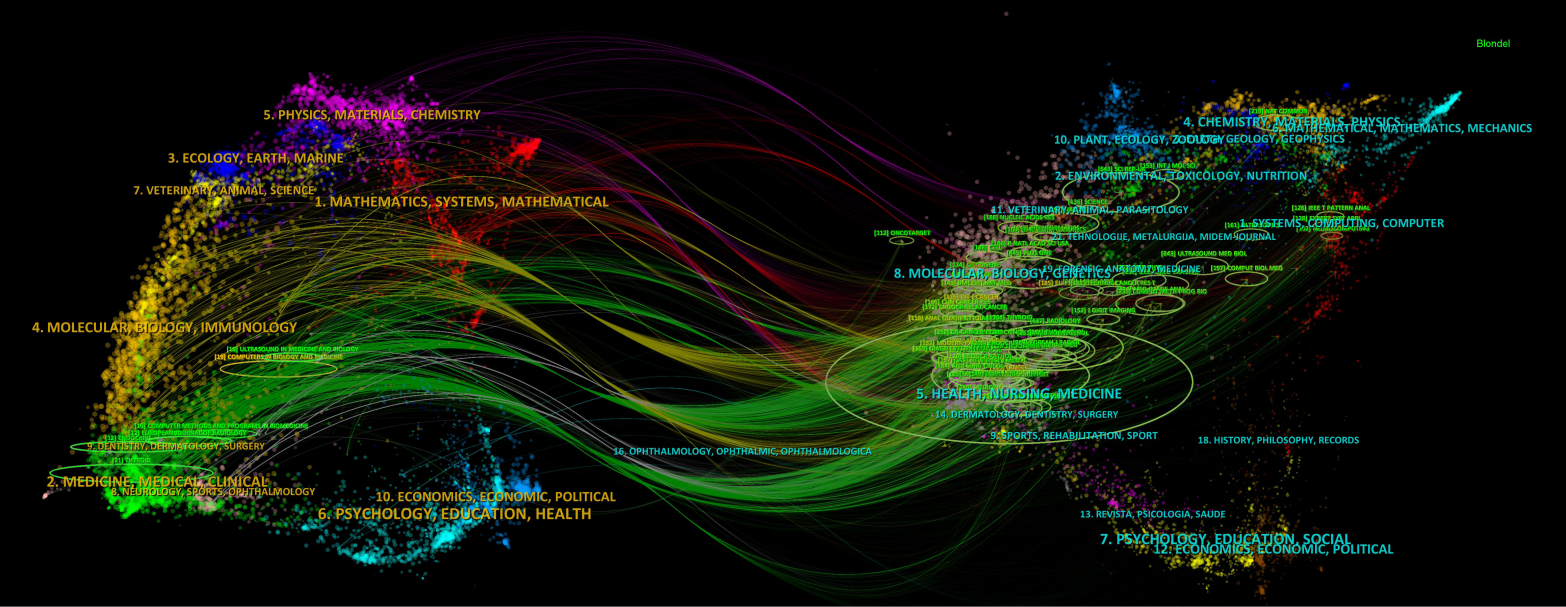
The journal dual-map overlay concerning research on the application of artificial intelligence in thyroid cancer.

The dual-map overlay (**Figure 5**), generated using CiteSpace, illustrates the citation relationships between journals and co-cited journals. The colored paths from left to right represent citation trajectories, with the left side denoting the citing journal areas and the right side representing the cited journal areas. The figure features yellow and blue words against a backdrop with aurora-like colors. The yellow and blue clusters represent distinct research themes or fields of study, with yellow focusing on a specific set of research areas, while the blue cluster highlights another. The aurora-like colors in the background represent the density and interconnectedness of these research areas, with more intense colors indicating areas of higher citation activity. The varying shades indicate how frequently certain terms or research themes appear together in the literature, reflecting their relevance and influence within the broader academic community. The journals publishing these articles are predominantly in the medical field, encompassing areas such as surgery, oncology, imaging, and medical engineering. The citing journals primarily originate from fields such as oncology and endocrinology, while the cited journals predominantly belong to the domains of radiology and computer science. The current field is flourishing, with its research foundation rooted in a broad spectrum of literature, rather than being limited to a single category or a few specific areas. This underscores the field’s interdisciplinary nature. For further advancement, it will require collaborative efforts from multiple disciplines, such as fostering partnerships between computer science and medicine.

### Analysis of authors and co-cited authors

**Figure 6:**
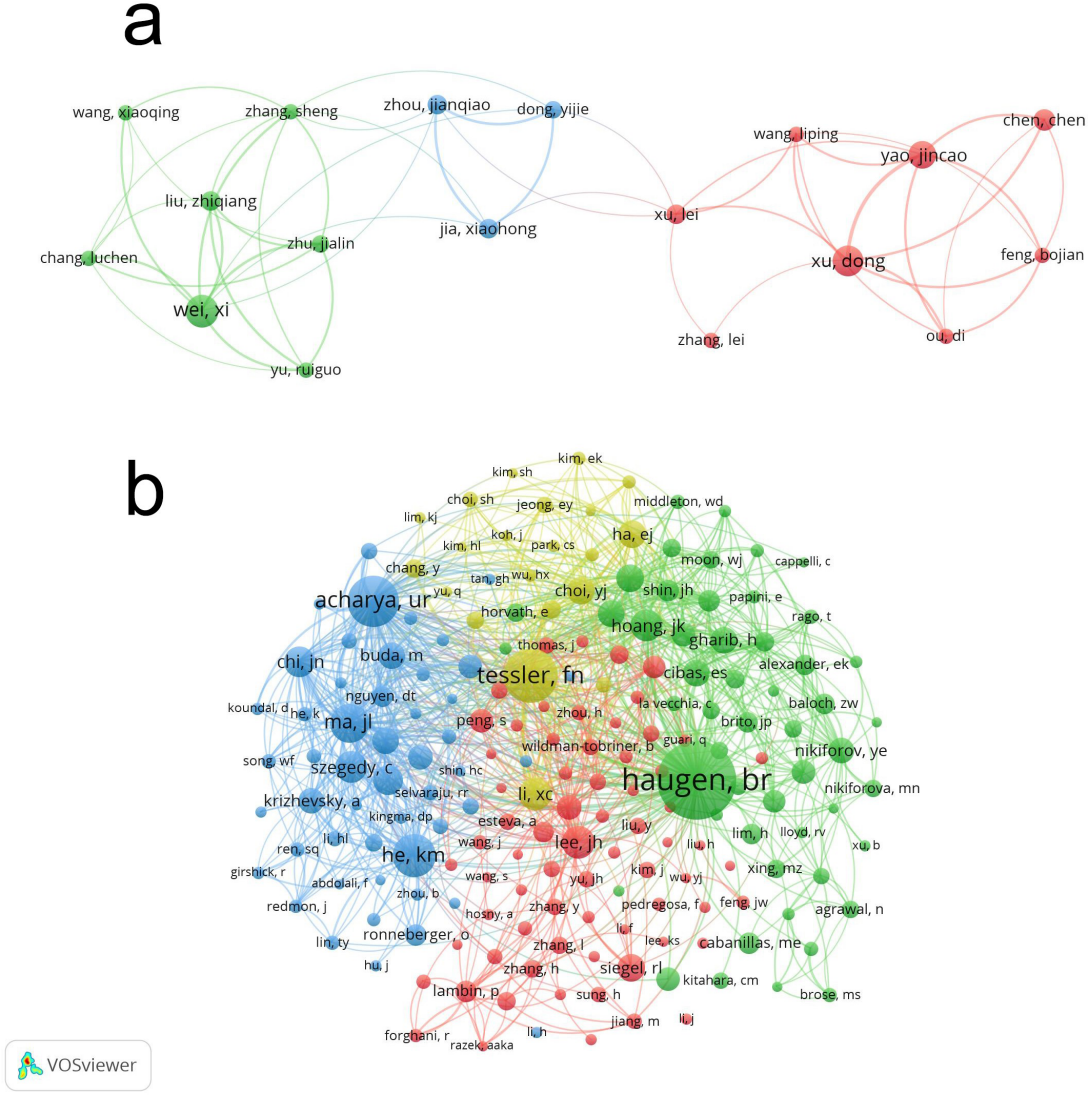
Visualization of authors **(A)** and co-cited Authors **(B)** on application of artificial intelligence in thyroid cancer.

An author’s number of published works serves as a key indicator of their influence and scholarly impact within the respective field (13). A total of 5,604 authors have published research on the application of artificial intelligence in thyroid cancer. **Table 3** lists the top 10 authors by publication count, each of whom has authored at least 6 papers in this field. Wei, X. has published 14 articles, which have been cited 413 times. He holds the highest number of publications as well as the greatest citation count. We employed VOSviewer to map the collaborative relationships among the authors (**Figure 6a**), which included 42 authors, each of whom had published no fewer than five related articles. Articles with more than 25 authors were automatically excluded.

**Table 3:** Top 10 authors and co-cited authors on research of application of artificial intelligence in thyroid cancer.

Rank	Authors	Country	Count	Citations	Co-cited authors	Citations	Country
1	Wei, X.	China	14	413	Haugen, B. R.	297	USA
2	Xu, D.	China	13	65	Tessler, F. N.	182	USA
3	Yao, J. C.	China	11	48	Acharya, U. R.	167	India
4	Chen, C.	China	8	29	He, K. M.	133	China
5	Jia, X. H.	China	7	209	Ma, J. L.	115	China
6	Liu, Z. Q.	China	7	79	Lee, J. H.	94	South Korea
7	Xu, L.	China	7	62	Li, X. C.	93	China
8	Zhou, J. Q.	China	7	234	Hoang, J. K.	90	USA
9	Dong, Y. J.	China	6	78	Szegedy, C.	89	USA
10	Zhu, J. L.	China	6	89	Chi, J. N.	87	China

In the co-cited author network analysis, a total of 22,350 co-cited authors were identified, among whom six authors were co-cited at least 100 times (**Table 3**). The top three most frequently cited authors were Haugen, B.R. (n=297), Tessler, F. N. (n=182) and Acharya, U. R. (n=167). In addition, a co-citation analysis was conducted to illustrate the operational and thematic influences of 192 interconnected authors who have been cited more than 20 times (**Figure 6b**). Haugen, B.R., Tessler, F. N. and Acharya, U. R., among others, exhibit strong collaborative relationships.

### Analysis of co-cited references

**Figure 7:**
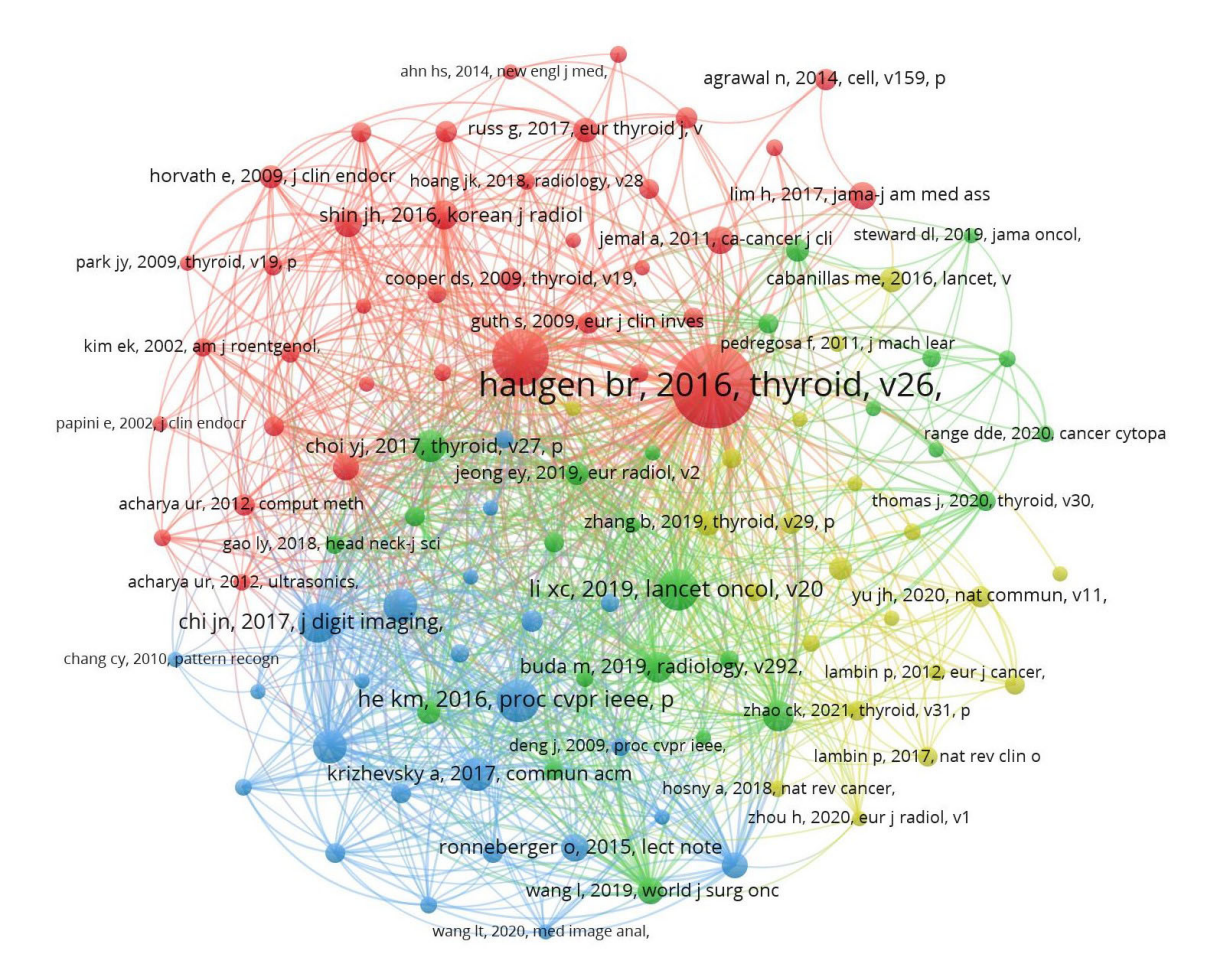
The visualization of co-cited references on research of application of artificial intelligence in thyroid cancer.

To explore the key topics and core literature on artificial intelligence in thyroid cancer, we analyzed 31,308 co-cited references over the past two decades. **Table 4** indicates that two references (14, 15) have been co-cited more than 100 times. The visualization in **Figure 7**, generated using VOSviewer, depicts a co-citation network analysis derived from 107 references, each of which has been co-cited a minimum of 20 times. Notable references such as ‘Haugen BR, 2016, Thyroid,’ ‘Tessler FN, 2017, J Am Coll Radiol,’ and ‘He KM, 2016, proc cvpr ieee’ exhibit strong co-citation relationships.

**Table 4:** Top 10 co-cited references on application of artificial intelligence in thyroid cancer.

Rank	Co-cited reference	Main research content	Citations
1	Haugen Br, 2016, Thyroid, V26, P1 (1)	2015 American thyroid association management guidelines for adult patients with thyroid nodules and differentiated thyroid cancer	268
2	Tessler Fn, 2017, J Am Coll Radiol, V14, P587 (2)	ACR TI-RADS Committee’s recommendations for managing thyroid nodules based on ultrasound appearance	146
3	He Km, 2016, Proc Cvpr Ieee, P770 (3)	Deep residual learning for image recognition	98
4	Li Xc, 2019, Lancet Oncol, V20, P193 (4)	Diagnosis of thyroid cancer using deep convolutional neural network models based on ultrasound imaging	90
5	Chi Jn, 2017, J Digit Imaging, V30, P477 (5)	Thyroid nodule classification in ultrasound images by fine-tuning deep convolutional neural network	84
6	Simonyan K, 2015, ArXiv (6)	Very deep convolutional networks for large-scale image recognition	67
7	Krizhevsky A, 2017, Commun ACM, V60, P84 (7)	ImageNet classification with deep convolutional neural networks	66
8	Ma Jl, 2017, Ultrasonics, V73, P221 (8)	A pre-trained convolutional neural network-based method for thyroid nodule diagnosis	65
9	Choi Yj, 2017, Thyroid, V27, P546 (9)	Deep learning-based computer-aided diagnosis system for localization and diagnosis of metastatic lymph nodes on ultrasound	63
10	Peng S, 2021, Lancet Digit Health, V3, PE250 (10)	Deep learning-based artificial intelligence model to assist thyroid nodule diagnosis and management	59

### Reference with citation bursts

**Figure 8:**
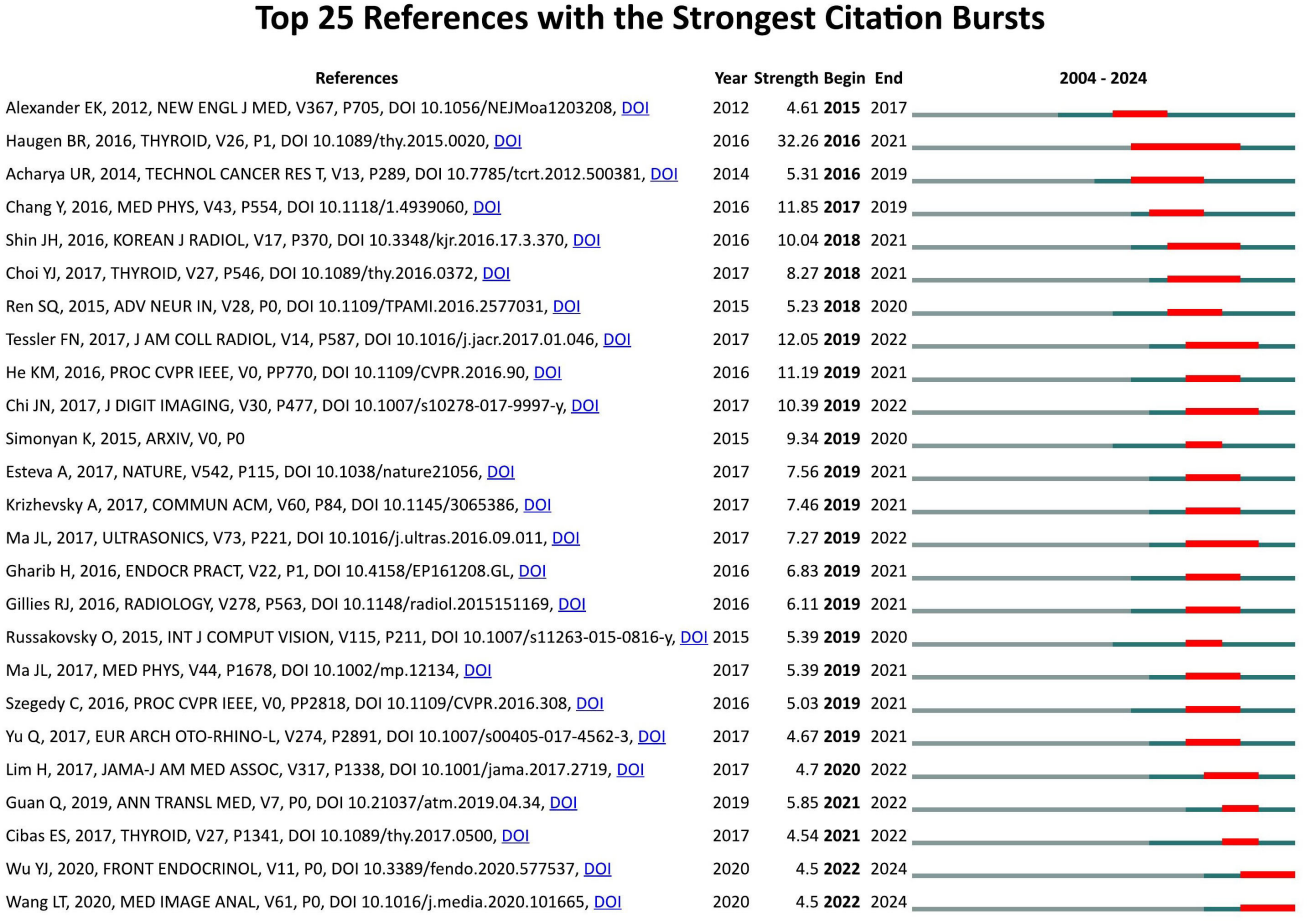
Top 25 references with strong citation bursts.

The phenomenon where a particular publication experiences a sudden surge in citations within a specific time period is referred to as a “reference with citation burst.” This occurrence typically indicates that the research content of the publication has become highly significant or relevant within its field, often due to the emergence of new technologies, discoveries, or trending research topics. Using CiteSpace, we identified the top 25 references with the highest citation bursts (**Figure 8**). The red bars represent periods of strong citation bursts from 2004 to 2024. The earliest citation burst occurred in 2015, while the latest was observed in 2022. The reference with the strongest citation burst (strength = 32.26) is ranked 2nd in **Table 5**, titled ‘2015 American Thyroid Association Management Guidelines for Adult Patients with Thyroid Nodules and Differentiated Thyroid Cancer: The American Thyroid Association Guidelines Task Force on Thyroid Nodules and Differentiated Thyroid Cancer.’ (14) This is also the most co-cited reference in studies applying AI to thyroid cancer. It was published by Haugen, B. R., et al. in *Thyroid*, with a citation burst lasting for four years (2016–2021). **Table 5** provides detailed information on the 25 references, listed in the order of publication shown in **Figure 8**, with burst strengths ranging from 4.5 to 32.26 and durations lasting 3 to 5 years.

**Table 5:** The main research contents of the 25 references with strong citations bursts.

Rank	Strength	Main research content
1	4.61	Preoperative diagnosis of benign thyroid nodules with indeterminate cytology (11)
2	32.35	2015 American thyroid association management guidelines for adult patients with thyroid nodules and differentiated thyroid cancer (1)
3	5.31	A review on ultrasound-based thyroid cancer tissue characterization and automated classification (12)
4	11.85	Computer-aided diagnosis for classifying benign versus malignant thyroid nodules based on ultrasound images (13)
5	10.04	Ultrasonography diagnosis and imaging-based management of thyroid nodules (14)
6	8.27	A computer-aided diagnosis system using artificial intelligence for the diagnosis and characterization of thyroid nodules on ultrasound (15)
7	5.23	Towards real-time object detection with region proposal networks (16)
8	12.05	ACR TI-RADS committee’s recommendations for managing thyroid nodules based on ultrasound appearance (2)
9	11.19	Deep residual learning for image recognition (3)
10	10.39	Thyroid nodule classification in ultrasound images by fine-tuning deep convolutional neural network (17)
11	9.34	Very deep convolutional networks for large-scale image recognition (6)
12	7.56	Dermatologist-level classification of skin cancer with deep neural networks (18)
13	7.46	ImageNet classification with deep convolutional neural networks (19)
14	7.27	Thyroid nodule classification in ultrasound images by fine-tuning deep convolutional neural network (5)
15	6.83	American Association of Clinical Endocrinologists, American College of Endocrinology, and Associazioe Medici Endocrinologi medical guidelines for clinical practice for the diagnosis and management of thyroid nodules (20)
16	6.11	The process of radiomics (21)
17	5.39	The ImageNet Challenge drives advances in object recognition with a large dataset, breakthroughs, and future directions
18	5.39	Cascade convolutional neural networks for automatic detection of thyroid nodules in ultrasound images (22)
19	5.03	The inception architecture for computer vision (23)
20	4.67	Computer-aided diagnosis of malignant or benign thyroid nodes based on ultrasound images (24)
21	4.7	Trends in thyroid cancer incidence and mortality in the United States (25)
22	5.85	Deep learning based classification of ultrasound images for thyroid nodules (26)
23	4.54	A pre-trained convolutional neural network-based method for thyroid nodule diagnosis (8)
24	4.5	Machine learning algorithms for the prediction of central lymph node metastasis in patients with papillary thyroid cancer (27)
25	4.5	Automatic diagnosis for thyroid nodules in ultrasound images by deep neural networks (28)

### Keyword and trend topic analysis

Co-occurrence analysis of keywords helps reveal the main areas of focus and emerging trends in AI-related research on thyroid cancer. Among a total of 3,522 keywords, we summarized the common keywords in this field (**Table 6**). Representative keywords include ‘cancer,’ ‘ultrasound,’ ‘management,’ ‘diagnosis,’ ‘deep learning,’ and ‘risk,’ indicating that these topics are research hotspots in the field.

**Table 6:** Top 30 keywords on research of application of artificial intelligence in thyroid cancer.

Rank	Keyword	Count	Rank	Keyword	Count
1	cancer	467	16	benign	71
2	ultrasound	220	17	risk	70
3	thyroid cancer	194	18	computer-aided diagnosis	58
4	management	177	19	radiomics	58
5	machine learning	172	20	thyroid	58
6	deep learning	164	21	fine-needle-aspiration	53
7	diagnosis	162	22	expression	51
8	thyroid nodule	153	23	segmentation	44
9	artificial intelligence	149	24	association	40
10	classification	149	25	prediction	39
11	nodules	130	26	risk stratification	39
12	association guidelines	91	27	model	38
13	system	79	28	data system	35
14	papillary thyroid carcinoma	73	29	images	34
15	features	72	30	ultrasound images	34

**Figure 9:**
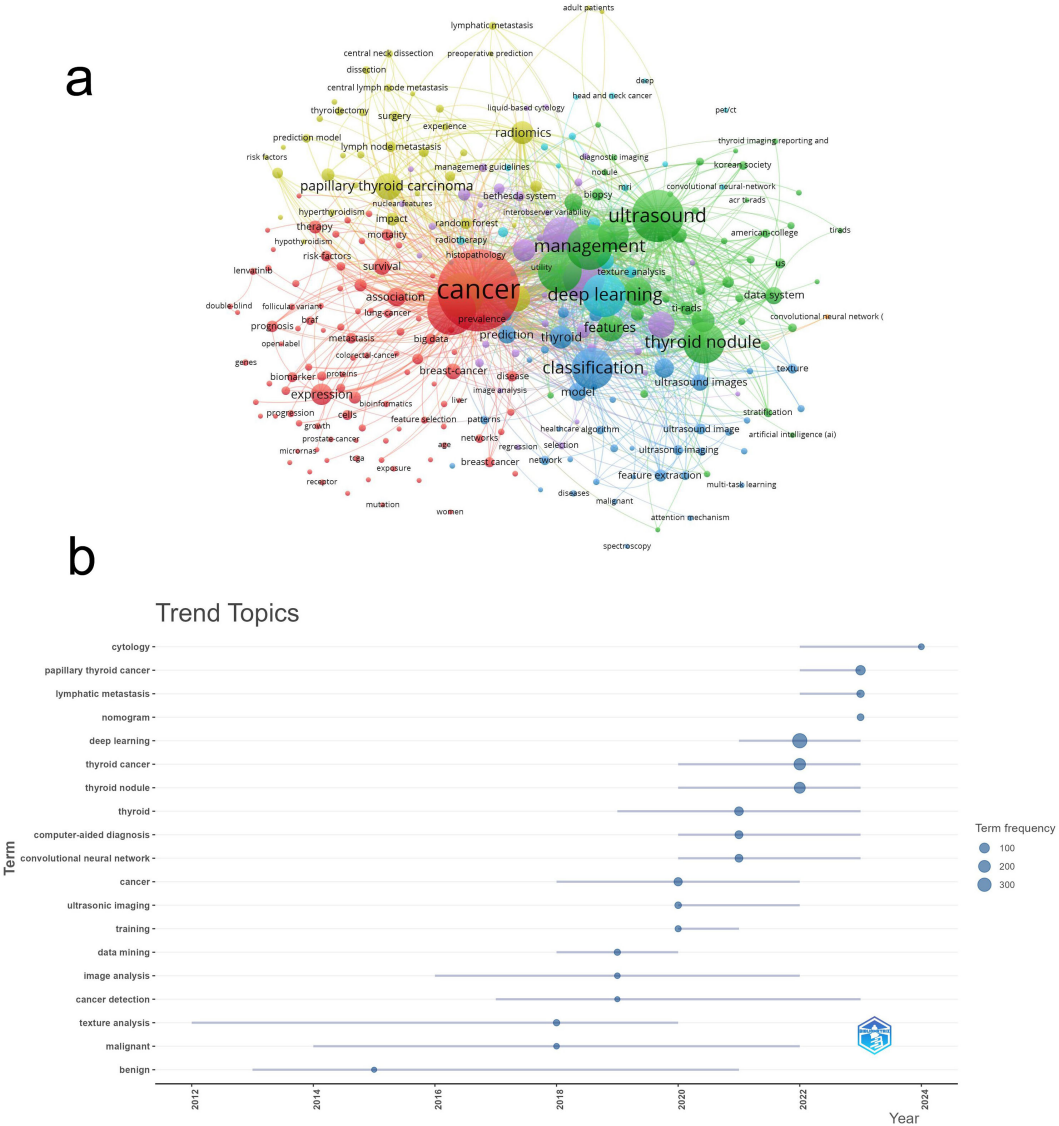
Keyword cluster analysis **(A)** and trend topic analysis **(B)**.

Using VOSviewer, we merged duplicate keywords and conducted a cluster analysis on 284 keywords that appeared at least five times. In the resulting network visualization map (**Figure 9a**), different colors represent different research directions. Seven main clusters were identified and are depicted in distinct colors. The green cluster includes terms such as ‘ultrasound’ and ‘diagnosis,’ primarily focusing on the use of AI in imaging analysis and disease diagnosis for thyroid cancer. The yellow and blue clusters represent specific subfields or interdisciplinary topics within AI in thyroid cancer research, with yellow focusing on terms related to ‘papillary thyroid carcinoma,’ ‘radiomics,’ and ‘survival prediction,’ while the blue cluster emphasizes ‘deep learning,’ ‘ultrasound imaging,’ and ‘classification models.’ The background aurora-like colors reflect the density and proximity of related keywords, visually indicating how closely these terms are associated with each other in the literature. The variation in color intensity is used to highlight the strength of the relationships between the keywords: brighter areas indicate stronger associations, while more subdued colors represent less frequent co-occurrence of terms.

According to the thematic analysis of keyword trends (**Figure 9b**), early research on the application of artificial intelligence in the field of thyroid cancer primarily focused on image analysis. Up until 2020, there was considerable attention on leveraging AI, particularly through ultrasound and other imaging modalities, to enhance the accuracy of thyroid cancer diagnoses. In recent years, deep learning and lymph node metastasis have also emerged as prominent research topics. The appearance of ‘cytology’ as a trending keyword in 2024 suggests that AI’s potential in analyzing thyroid cancer at the cellular level is now beginning to be explored.

## Discussion

We collected 956 publications on artificial intelligence (AI) in the field of thyroid cancer research from the Web of Science Core Collection (WOSCC) database. Using visualization software such as VOSviewer and CiteSpace, we conducted a bibliometric analysis to gain a clear understanding of the current research progress in AI-assisted diagnosis and treatment of thyroid cancer, as well as to forecast future research hotspots and trends.

### General information

Over the past two decades, the application of AI in thyroid cancer research has evolved from a small number of publications each year to an exponential growth. Notably, over 70% of the publications in this field have emerged within the last five years. China leads in publication volume, contributing 50.84% of the total, although the quality of its research lags behind that of the United States, which ranks second in terms of publication volume. Among the top 10 institutions ranked by publication output, nine are based in China and one in South Korea. This phenomenon may be attributed to the higher incidence of thyroid cancer in Asia, particularly in China and South Korea (16, 17), which has fostered a greater interest in applying AI to thyroid cancer research.

Collaboration among Chinese institutions, such as Shanghai Jiao Tong University, the Chinese Academy of Sciences, and Zhejiang University, is particularly strong. However, partnerships with institutions from other countries remain limited. To accelerate advancements in the application of artificial intelligence in thyroid cancer and related fields, it is crucial to actively encourage cross-border collaboration among institutions worldwide. This will foster the sharing and optimization of research resources, ultimately speeding up the development of AI-driven innovations in thyroid cancer research and beyond.

The top three journals by publication volume are *Frontiers in Oncology*, *Frontiers in Endocrinology*, and *Scientific Reports*, each with more than 30 articles. Scholars in this field may consider prioritizing these journals for disseminating their findings. Notably, among the top ten journals ranked by co-citation frequency, L*ecture Notes in Computer Science (LNCS)* is not indexed in the Science Citation Index (SCI). Instead, it is a well-known conference proceedings series published by Springer, primarily covering research in computer science. arXiv is not a journal; it is an open-access preprint repository. Operated by Cornell University, arXiv allows researchers to upload their manuscripts prior to formal peer review, making them available for other scholars to read and comment on. It covers a range of fields, including physics, mathematics, computer science, statistics, engineering, and biology.

China’s Wei, X. is the most prolific author in this field, with 14 publications, primarily focusing on developing deep learning models based on ultrasound images for thyroid nodule diagnosis (18). Notably, all of the top ten authors by publication volume are from China, reflecting the country’s strong interest and significant research achievements in applying artificial intelligence to thyroid cancer research. This may be related to the previously mentioned high incidence of thyroid cancer in China. The most frequently co-cited author is Haugen, B. R. from the United States, who is the principal author of the 2015 American Thyroid Association Management Guidelines for Adult Patients with Thyroid Nodules and Differentiated Thyroid Cancer (14).

### Knowledge base

A co-cited reference refers to a situation where two or more articles are cited together in other papers or publications. Essentially, it indicates that these references are frequently cited together by other researchers in the same or related fields. This co-citation relationship can highlight how certain studies or concepts are linked or have influenced the development of specific areas of research. Co-citation analysis is often used in bibliometrics to map scientific literature, showing how different works are related through the patterns of citations they receive. An analysis of the ten most frequently co-cited references reveals that the current research foci on the application of artificial intelligence in thyroid cancer predominantly center on deep learning in diagnostics (19, 20). In diagnosis, the primary focus is on developing convolutional neural network models based on ultrasound and other imaging modalities (21).

### Research hotspots and outlook

References with citation bursts can assist researchers in identifying works that have had a significant impact on the field during specific time periods. Based on the content of the 25 references with citation bursts (**Table 4**), image analysis for auxiliary diagnosis has been a hotspot in the application of artificial intelligence in thyroid cancer since 2010. This trend is further supported by the co-occurrence analysis of keywords. The primary keywords include ‘diagnosis,’ ‘deep learning,’ ‘ultrasound,’ and ‘lymphatic metastasis.’ In conjunction with the trend topic analysis, we suggest that future research on AI in thyroid cancer should focus on the following key areas.

Artificial intelligence-based image recognition will remain a major focus of research for the foreseeable future. Significant advancements have been made in the application of artificial intelligence (AI) in the analysis of medical images for thyroid cancer, particularly through the use of deep convolutional neural networks (DCNN) to analyze ultrasound, CT, and MRI data. In the realm of ultrasound imaging, AI technology has demonstrated its capability to accurately differentiate between benign and malignant thyroid nodules, as well as predict cancer differentiation, malignancy, and lymph node metastasis (19, 22, 23). In CT and MRI imaging, AI has been employed to assess lesion size, morphology, and tissue type, with the added advantage of more refined analysis through 3D convolutional neural networks (3D-CNN) (24–27). The integration of multimodal imaging data has further enhanced diagnostic accuracy by combining various types of imaging information, thus improving the precision of thyroid cancer diagnosis. While there are also studies involving model training with histopathological images (28–30), their number is relatively limited. It is important to note, however, that deep learning model training relies on large volumes of high-quality medical data. The current lack of standardized data across different hospitals restricts the generalizability of these models. This is particularly evident with ultrasound images, which are heavily dependent on the skill level of the operator. Variations in experience among physicians result in differing ultrasound image quality, ultimately affecting model accuracy. Some studies have begun training models using dynamic ultrasound videos (31), which partially addresses this issue. Nevertheless, the development of more advanced AI technologies remains crucial—technologies that can train highly accurate models with smaller datasets or leverage unsupervised learning to achieve automatic data labeling and recognition.

In addition to incorporating radiologic and pathologic images, artificial intelligence can integrate various data sources—such as patient demographics, treatment plans, and genomic information—to predict the risk of postoperative and metastasis (32–34). By identifying prognostic factors that may influence outcomes and combining them with individual patient profiles, AI can offer personalized treatment recommendations for physicians, thereby advancing the development of precision medicine. To advance the development of precision medicine, these studies often rely on clinical data and imaging records from medical centers, as well as the close collaboration between AI/machine learning researchers and clinical practitioners. The efficacy of AI models is intimately linked to the scale and quality of the datasets utilized. Some studies (26, 35, 36) are based solely on small datasets comprising a few dozen patient records, which are typically used for preliminary validation or investigations into specific, narrowly defined populations. However, the limitations of such datasets are significant, as they fail to adequately represent a broader patient population. In contrast, other studies (37–39) leverage large-scale datasets derived from multiple medical centers, encompassing thousands or even tens of thousands of patient records. These extensive datasets enable AI models to achieve superior generalization, adapt to diverse patient populations across various regions and institutions, and enhance the reliability and accuracy of the models in clinical applications.

Recent research in cytology represents an emerging focal point in the application of artificial intelligence to thyroid cancer. One notable study developed a deep-learning algorithm specifically designed to analyze whole-slide images (WSIs) from fine-needle aspiration biopsies (FNAB) of the thyroid (40). By analyzing the morphology of thyroid cancer cells, gene expression patterns, and the associated cellular microenvironment, artificial intelligence holds vast untapped potential for further applications in the field of thyroid cancer cytology (36, 41, 42).

In conclusion, AI in the field of thyroid cancer is still in its early stages, with numerous potential applications yet to be realized. As technology advances and public acceptance of AI increases, AI will likely see widespread use in the clinical management of thyroid cancer.

### Limitations

However, our study was not without limitations. First, our reliance solely on the Web of Science Core Collection means that some relevant publications indexed in other databases, such as PubMed and Embase, may have been overlooked. Second, the study only included articles published in English, potentially excluding important research published in other languages. Nevertheless, we believe that the study covers the vast majority of relevant publications, and despite these omissions, the conclusions drawn from the data remain robust and valid.

## Conclusion

This study employs bibliometric analysis to delve into the body of literature on thyroid cancer and artificial intelligence (AI) published over the past two decades. AI has witnessed rapid development in the field of thyroid cancer, particularly in the application of deep learning techniques for diagnostic assistance and prognostic model prediction. These areas represent key focal points of current research and are likely to remain so in the foreseeable future. China has placed significant emphasis on this domain of study; however, greater collaboration with other countries and a concerted effort to enhance research quality are essential.
